# Evaluation of the significance of interleukin-6 in the diagnosis of postoperative pneumonia: a prospective study

**DOI:** 10.1186/s12872-022-02744-0

**Published:** 2022-07-07

**Authors:** Wei Chen, Kai Zhong, Yan Guan, Hai Tao Zhang, He Zhang, Tuo Pan, Jun Pan, Dong Jin Wang

**Affiliations:** 1 Department of Cardio-Thoracic Surgery, Nanjing Drum Tower Hospital Clinical College of Nanjing Medical University, Nanjing, 210008 Jiangsu China; 2 Department of Cardio-Thoracic Surgery, Nanjing Drum Tower Hospital, The Affiliated Hospital of Nanjing University Medical School, Nanjing, 210008 Jiangsu China; 3 Department of Cardio-Thoracic Surgery, Nanjing Drum Tower Hospital, Peking Union Medical College, Chinese Academy of Medical Sciences, Graduate School of Peking Union Medical College, Beijing, 100010 China

**Keywords:** Pneumonia, Interleukin-6, Cardiac surgery

## Abstract

**Background:**

Postoperative pneumonia (PP) is one of the most common complications after cardiac surgery. This study was designed to access the diagnostic value of interleukin-6 (IL-6) for pneumonia within the first 5 days after cardiac surgery in adults.

**Method:**

This prospective observational study enrolled 694 patients who admitted to our center from 10 October 2020 to 30 June 2021. Blood samples were collected after admission and on five consecutive days after surgery to measure IL-6, procalcitonin (PCT), C-reactive protein (CRP) and white blood cells (WBC) respectively. Combined with clinical data, we assessed the diagnostic performance of different biomarkers using univariate and multifactorial analyses as well as receiver operating characteristic curves (ROC) and the area under the curve (AUC).

**Result:**

Finally, 68 patients were diagnosed with PP (PP Group). In addition, 626 cases were assigned to the control group (Non-PP Group). From postoperative day 1 (POD1) to day 5, IL-6 and PCT levels showed higher diagnostic value (P < 0.001, P < 0.05, respectively); meanwhile, there was no difference in white blood cell counts between the two groups; CRP showed some value from POD2 onwards (P < 0.001). Among these biomarkers, IL-6 on POD1 [AUC: 0.78, 95% confidence interval (CI): 0.71–0.83], IL-6 on POD2 (AUC: 0.77, 95% CI: 0.71–0.82) and CRP levels on POD3 (AUC: 0.77, 95% CI: 0.70–0.84) had the highest diagnostic value. Multivariate analysis found that smoking status [odds ratio(OR): 7.79, 95% CI: 3.05, 19.88, p < 0.001], drinking status (OR: 22.68, 95% CI: 9.29, 55.37, p < 0.001) and hypertension (OR: 2.85, 95% CI: 1.28, 6.35, p = 0.011), IL-6 on POD2 (OR: 1.01, 95% CI: 1.00, 1.01, p = 0.018), mechanical ventilation time (OR: 1.03, 95% CI: 1.00, 1.05, p = 0.040) and intensive care unit stay time (OR: 1.01, 95% CI: 1.00, 1.02, p < 0.001) were independent risk factors for postoperative pneumonia.

**Conclusion:**

Smoking, drinking, hypertension, prolonged duration of mechanical ventilation and intensive care unit stay, and IL-6 on POD2 were independent risk factors for pneumonia after cardiovascular surgery. IL-6 level on POD2 may serve as a promising indicator, better than WBC, PCT and CRP.

## Background

Postoperative pneumonia (PP) is one of the most common and serious complications after cardiac surgery[1–4]. The incidence of PP is associated with increased mortality, readmission rate, prolonged hospitalization and higher costs[3–5]. Therefore, early diagnosis of PP may contribute to clinical decision-making and improve prognosis.

In clinical practices, we routinely used the White blood cell (WBC) count, procalcitonin (PCT) and C‐reactive protein (CRP) to predict postoperative infections[6–10]. However, both cardiac surgery and cardiopulmonary bypass (CPB) may lead to acute inflammation in the first few days after operation[9, 11]. During this period, the commonly used biomarkers mentioned above may be misleading or ineffective[12, 13].

Interleukin-6 (IL-6) plays a key role in both immune and inflammatory responses[4, 14–16]. In the process of infection and tissue injury, IL-6 responds more rapidly than WBC and CRP[8]. And, IL-6 could be used as an efficient biomarker for the diagnosis of pneumonia[14, 16]. However, there are insufficient studies to support the value of IL-6 for the diagnosis of pneumonia in the first few days after cardiac surgery in adults.

We therefore conducted this study to assess the value of IL-6 in the diagnosis of early pneumonia after cardiac surgery and compare it with that of PCT, CRP and WBC counts.

## Methods

### Study design and patients

This prospective observational study was conducted in the Department of Cardiothoracic surgery, Nanjing Drum Tower Hospital and approved by Medical Ethics Committee of Affiliated Nanjing Drum Tower Hospital, Nanjing University Medical College (2020–249-01). All patients enrolled provided written informed consent. A total of 694 patients who underwent cardiac surgery in our center from October 10, 2020 to June 30, 2021 were enrolled. The inclusion criteria were (1) adult patients between 18 and 80 years old; (2) patients scheduled to undergo cardiac surgery with CPB; (3) written informed consent to participate in the study. Exclusion criteria were (1) preoperative body temperature ≥ 38 ℃ or white blood cell count > 10,000 cells/mm3 or serum IL-6 > 10 pg/ml; (2) patients with ongoing infective endocarditis, pneumonia or any infectious diseases; (3) patients with inflammatory immune diseases or connective tissue diseases; (4) pregnant or lactating women; (5) patients refuse to take part in this study.

### Perioperative management

We routinely use prophylactic antibiotic therapy to prevent infection during the perioperative period. The first dose of antibiotics was given intravenously within 60 min of the incision and the antibiotics were administered continuously every 3–4 h during the operation[17]. In addition, according to the recommendations of the guidelines, we continued to use adequate antibiotics for the first 48 h after the operation. The first-generation cephalosporins were our routine preventive intravenous antibiotics, and vancomycin could be used when clinicians consider it necessary[18].

### Diagnosis of PP and group

PP will be diagnosed when the patient meets both clinical and bacteriological strategies. The clinical strategy means the presence of a new or progressive radiographic infiltrate plus at least two of three clinical features (fever greater than 38 °C, leukocytosis or leucopenia and purulent secretions). The bacteriological strategy is as follows: Sputum culture was performed at least twice with fiberoptic bronchoscope or alveolar lavage fluid. Pathogenic bacteria were detected in sputum culture and the first positive result appeared within 48 h after surgery[19]. Pleural effusion culture examination was performed to find pathogens when necessary.

The diagnosis of PP was determined by two independent experts based on complete clinical records. A third expert reached a consensus in cases of disagreement between the two clinical experts. Each final diagnosis was classified as microbiologically confirmed, probable, possible, or absent. Experts were blinded for IL-6 but not for WBC and CRP. The final diagnosis was reached when it was classified as microbiologically confirmed by the experts and ruled out when the experts classified it as absent, probable or possible[9].

Patients were eventually divided into two groups: the non-PP group, who did not develop pneumonia within 5 days after surgery; and the PP group, who developed pneumonia within 5 days after surgery.

### Laboratory measurements

Blood samples were collected after admission and on five consecutive days after surgery to measure IL-6, procalcitonin (PCT), C-reactive protein (CRP) and white blood cells (WBC) respectively. PCT was measured by an immune luminometric assay whose detection limit range from 0.05 to 25 ng/ml and normal value was less than 0.5 ng/ml. CRP was measured by automatic laser nephelometry with a normal value less than 8 mg/L. WBC was determined by using an automatic counter with a normal value between 4,000 and 10,000 cells/mm3. IL-6 levels were determined by ELISA, with capture and peroxidase-labeled tracer antibody with a normal value less than 10 pg/mL. All patients were treated with bronchoalveolar lavage and sputum culture under fiberoptic bronchoscope on POD1-3.

### Data collection

Data were recorded prospectively. We collected all patients' data including demographic characteristics, preoperative diagnosis, surgical methods, CPB time, aortic cross clamp time, mechanical ventilation time, intensive care unit stay time, imaging examination results, and microbiological examination results through the electronic case system. Serum IL-6, CRP, PCT and WBC concentrations were recorded daily within the first 5 days after operation.

### Sample size

In our study, the diagnostic value of different biomarkers was evaluated based on the receiver operating characteristics (ROC) curves and area under the curve (AUC). Therefore, we used the Power Analysis and Sample Size (version 15.0) to calculate the required sample size based on AUC. In previous study, the AUC of IL-6 to diagnose infection ranged 0.75–0.85[14, 15]. So, we set the AUC of this study to 0.75. Considering a two-sided significance level of 5% (α) and confidence interval width 0.15 and a test power of 80% (1-β), 58 patients with PP were needed. Our study included 68 patients with pneumonia, which met the sample size requirement.

### Statistical analysis

All statistical analyses were conducted by SPSS version 26.0 (SPSS, IBM Corporation, NY). Continuous variables were presented as median and interquartile range (IQR), compared by the Mann–Whitney U test. Qualitative data were described as number (percentage). Categorical variables were presented as absolute numbers and percentages, equated by the chi-square test or Fisher’s exact test.

Receiver operating characteristics (ROC) curves and area under the curve (AUC) were used to evaluate the value of WBC, CRP, PCT and IL-6 in diagnosing PP. Cutoff values for WBC, CRP, PCT and IL-6 were chosen to correspond to the best respective Youden’s index calculated as follows: Youden’s index = sensitivity + specificity—1. Results are expressed for AUC as mean and 95% Confidence interval (CI). For additional analyses, all covariates (age, preoperative left ventricular ejection fraction ≤ 40%, smoking, drinking, hypertension, CPB time, aortic cross clamp time, mechanical ventilation time, intensive care unit stay time, hospital stay time, IL-6 on POD1-5, CRP on POD2-5, PCT on POD1-5) in univariate modeling were entered into a multivariable logistic regression model designed to assess the independent associated post-operative factors with PP. Collinearity diagnostics were performed using tolerance estimates for individual variables in a linear regression model. All reported P values are two sides. P < 0.05 was considered significant.

## Results

### Patient characteristics

694 patients were included in the study. 68 patients were diagnosed with PP (PP group) and 626 patients were categorized into the Non-PP group. No significant differences were found regarding age, sex, BMI, type of surgery, diabetes mellitus, previous surgery, CPB time, aortic cross clamp time, hospital stay time. There were significant differences between the two groups for history of smoking (P < 0.001), drinking (P < 0.001), hypertension (P = 0.037) and preoperative left ventricular ejection fraction ≤ 40% (P = 0.019). Patients in the PP group experienced a longer duration of mechanical ventilation (p < 0.001) and intensive care unit stay (p < 0.001) compared to the non-PP group. And, patients in PP group experienced a higher mortality (p = 0.008) (Shown in Table 1).

**Table 1:** Clinical baseline data of Patients in the PP and Non-PP Groups

Variable	PP Group (n = 68)	Non-PP Group (n = 626)	P
Age (year)	58.0 (52.0,69.8)	57.0 (49.0,67.0)	0.090
Gender Male (n,%)	46 (67.7)	364(58.1)	0.130
BMI	23.7(21.9,25.5)	24.1(22.0,26.4)	0.397
LVEF ≤ 40% (n,%)	12 (17.6)	55(8.8)	0.019
Type of cardiovascular surgery			0.352
Congenital heart surgery	1	19	
Valve surgery	22	287	
CABG	8	96	
CABG + Valve surgery	9	15	
Aortic surgery	1	105	
Aortic + Valve surgery	8	68	
Others	19	36	
Smoking status (n,%)	30(44.1)	67(10.7)	< 0.001
Drinking status (n,%)	43(63.2)	101(16.1)	< 0.001
Hypertension (n,%)	38(55.9)	260(41.5)	0.037
Diabetes mellitus (n,%)	8(11.8)	76(12.1)	0.865
Previous surgery (n,%)	24(35.3)	241(38.5)	0.491
Cardiopulmonary bypass time (min)	150.0 (117.5,181.0)	139.5 (105.0,185.8)	0.365
Aortic cross clamp time (min)	109.0 (84.3,136.5)	99.0 (70.0,135.0)	0.281
MV time (h)	22.5 (15.1,32.8)	13.5 (8.0,20.0)	< 0.001
Intensive Care Unit Stay time (h)	103.0 (72.0,237.3)	48.0 (28.6,72.0)	< 0.001
Hospital stay time (day)	16.0 (14.0,21.8)	16.0(13.0,19.0)	0.068
Death (n,%)	6(8.8)	17(2.7)	0.008

CABG Coronary artery bypass grafting, PP: Postoperative pneumonia, Median (interquartile range), n(%), MV Mechanical ventilation, LVEF Left ventricular ejection fraction

### WBC, CRP, PCT, and IL-6 in the two groups

**Fig.1:**
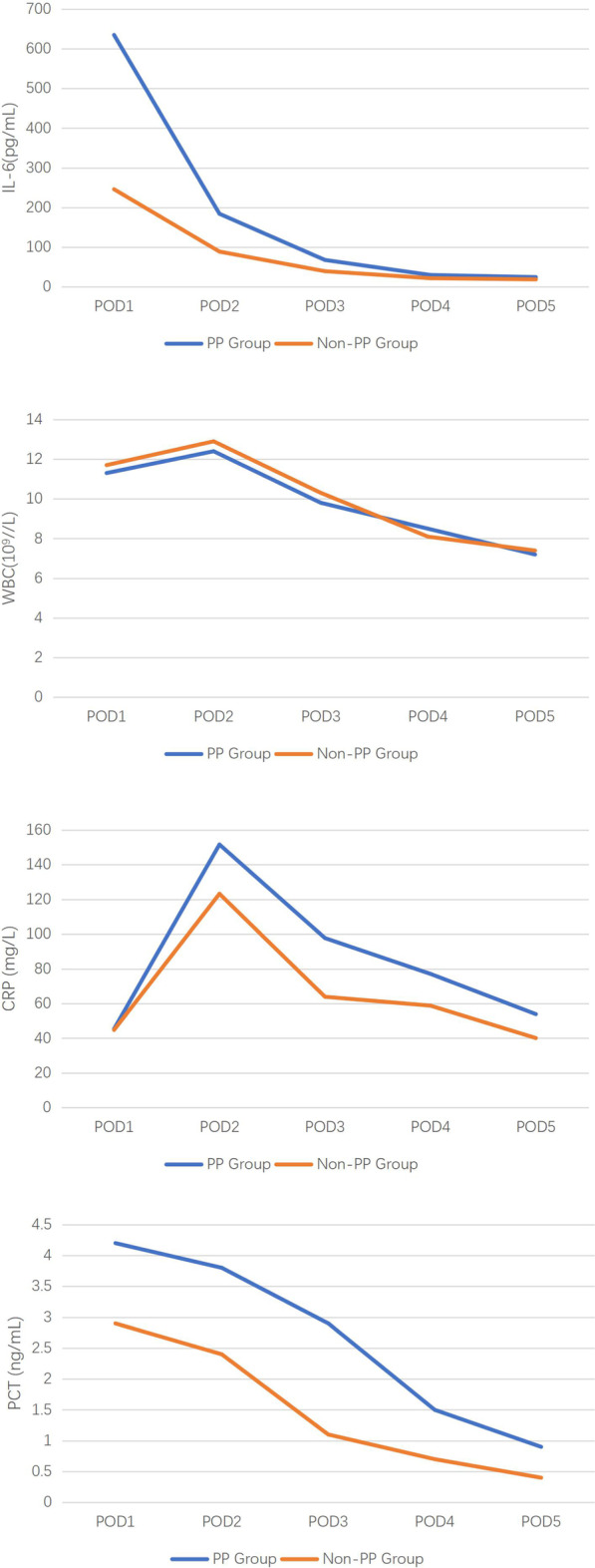
Comparison of serum IL-6, WBC, CRP and PCT in the PP (blue line; n = 68) and Non-PP (orange line; n = 626) groups. Data are expressed as median

**Fig.2:**
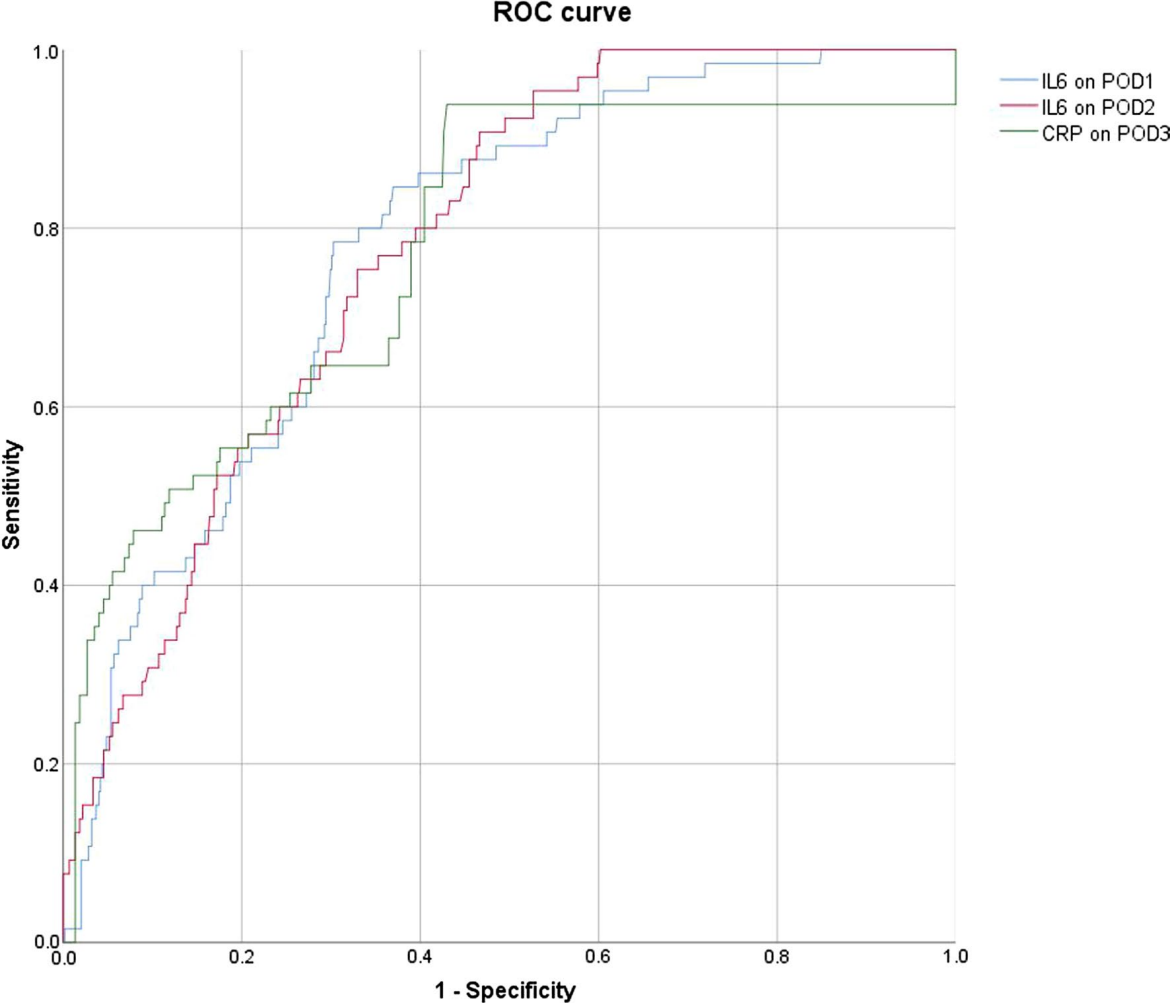
Comparison of the receiver operating characteristic curves and area under the curve showing the relation between sensitivity (true positive) and 1-specificity (true negative) in determining the value of IL-6 on POD1, IL-6 on POD2 and CRP on POD3 for the diagnosis of PP

There was no significant difference in WBC, CRP, PCT and IL-6 between the two groups at admission. After surgery, WBC peaked on POD2 and there was no difference between the two groups from POD1 to POD5. CRP levels peaked at POD2 and began to differ between the two groups (P < 0.001). From POD1 to POD5, both PCT and IL-6 levels were significantly higher in the PP group than in the non-PP group. (P < 0.05, P < 0.001, respectively) (Shown in Table 2 and Fig. 1). ROC curves showed that IL-6 levels on POD1 and POD2 and CRP on POD3 had the best diagnostic value (AUC > 0.75) (Shown in Table 3 and Fig. 2).

**Table 2:** Serum IL-6, WBC, CRP and PCT in the PP and Non-PP Groups

Variable	PP Group (n = 68)	Non-PP Group (n = 626)	P
IL-6(pg/mL)			
Preoperative	2.0 (1.5,5.7)	1.6 (1.5,5.5)	0.642
POD1	634.9 (370.0,1255.7)	246.0 (132.8,448.2)	< 0.001
POD2	184.2 (125.4,291.7)	88.9 (56.1,153.1)	< 0.001
POD3	67.8 (38.9,108.2)	39.5 (25.6,63.1)	< 0.001
POD4	29.9 (20.4,52.4)	21.8 (15.4,32.1)	< 0.001
POD5	24.6 (17.2,38.1)	18.9 (13.3,28.1)	< 0.001
WBC(10^9^//L)			
Preoperative	6.5 (5.4,8.2)	6.2 (5.1,8.0)	0.397
POD1	11.3 (9.4,13.4)	11.7 (9.8,14.4)	0.326
POD2	12.4 (9.8,15.5)	12.9 (10.6,15.7)	0.193
POD3	9.8 (8.0,12.4)	10.3 (8.3,12.7)	0.260
POD4	8.5 (6.8,9.9)	8.1 (6.7,10.1)	0.860
POD5	7.2 (6.2,10.0)	7.4 (6.3,9.3)	0.971
CRP (mg/L)			
Preoperative	0.9 (0.3,2.2)	0.7 (0.3,3.1)	0.758
POD1	45.4 (25.9,68.7)	44.8 (32.9,66.1)	0.465
POD2	151.6 (116.0,190.0)	123.2 (93.7,159.0)	< 0.001
POD3	97.7 (70.2,129.1)	63.9 (48.6,79.9)	< 0.001
POD4	77.1 (61.5,106.3)	58.8 (40.4,80.5)	< 0.001
POD5	53.9 (38.0,73.5)	40.1 (30.2,53.2)	< 0.001
PCT (ng/mL)			
Preoperative	0.04 (0.04,0.04)	0.04 (0.04,0.04)	0.545
POD1	4.2 (1.8,9.3)	2.9 (1.1,7.7)	0.020
POD2	3.8 (1.7,8.2)	2.4 (0.8,6.4)	0.002
POD3	2.9 (0.9,6.5)	1.1 (0.4,2.8)	< 0.001
POD4	1.5 (0.5,2.9)	0.7 (0.3,1.9)	< 0.001
POD5	0.9 (0.3,1.4)	0.4 (0.2,1.1)	0.001

Median (interquartile range), POD Postoperative day

**Table 3:** Comparison of AUC and Efficiency of IL-6,WBC, CRP and PCT in the Diagnosis of Postoperative Pneumonia

Variable	AUC (95%CI)	Cut-off	Sensitivity	Specificity
IL-6 (pg/ml)				
POD1	0.78 (0.71,0.83)	363.23	0.79	0.7
POD2	0.77 (0.71,0.82)	94.24	0.91	0.53
POD3	0.69 (0.61,0.76)	71.53	0.5	0.81
POD4	0.65 (0.57,0.72)	24.32	0.69	0.58
POD5	0.65 (0.57,0.72)	20.58	0.71	0.57
WBC (10^9^/L)				
POD1	0.45 (0.38, 0.53)	6.15	0.98	0.03
POD2	0.44 (0.36, 0.52)	23.45	0.05	0.99
POD3	0.43 (0.35, 0.50)	17.05	0.07	0.95
POD4	0.49 (0.41, 0.57)	8.85	0.44	0.62
POD5	0.48 (0.39, 0.56)	10.05	0.23	0.83
CRP (mg/L)				
POD1	0.48(0.39,0.56)	62.52	0.34	0.72
POD2	0.63(0.56,0.70)	133.93	0.66	0.59
POD3	0.77(0.70,0.84)	68.18	0.94	0.56
POD4	0.69(0.62,0.75)	51.23	0.91	0.41
POD5	0.67(0.60,0.74)	49.36	0.62	0.7
PCT (ng/mL)				
POD1	0.59(0.52,0.67)	3.28	0.65	0.54
POD2	0.61(0.53, 0.69)	3.3	0.62	0.6
POD3	0.68 (0.60, 0.75)	1.56	0.69	0.61
POD4	0.63 (0.55, 0.71)	0.86	0.69	0.55
POD5	0.63 (0.56, 0.70)	0.44	0.71	0.51

POD: Postoperative day

### Multivariate analysis between the PP and non-PP group

Multivariate Analysis found that smoking status [odds ratio(OR):7.79, 95% CI: 3.05, 19.88, p < 0.001], drinking status (OR:22.68, 95% CI: 9.29, 55.37, p < 0.001) and hypertension (OR: 2.85, 95% CI: 1.28, 6.35, p = 0.011), higher IL-6 on POD2 (OR: 1.01, 95% CI: 1.00, 1.01, p = 0.018), longer duration of mechanical ventilation time (OR: 1.03, 95% CI: 1.00, 1.05, p = 0.040) and intensive care unit stay (OR: 1.01, 95% CI: 1.00, 1.02, p < 0.001) were independent risk factors for PP. (Shown in Table 4).

**Table 4:** Multivariate Analysis Between the PP and the Non-PP Groups

Variable	OR(95%CI)	B	Wald	P
Smoking	7.79 (3.05,19.88)	2.05	18.43	< 0.001
Drinking	22.68 (9.29,55.37)	3.12	46.98	< 0.001
Hypertension	2.85 (1.28,6.35)	1.05	6.54	0.011
IL-6 on POD 2	1.01 (1.00,1.01)	0.01	5.64	0.018
Mechanical ventilation time	1.03 (1.00,1.05)	0.03	4.21	0.04
Intensive Care Unit Stay time	1.01 (1.00,1.02)	0.01	21.52	< 0.001

## Disgussion

Early diagnosis of PP is conducive to the rapid and appropriate formulation of reasonable treatment strategies, improve the prognosis of patients and save medical expenses[3, 11, 20]. Our study found that elevated IL-6 levels on POD2 as well as smoking, drinking, hypertension, prolonged mechanical ventilation and ICU stay were all independent risk factors for PP and that IL-6 may be a promising biomarker with good diagnostic value.

There was some literature suggesting that IL-6 rises earlier than PCT and CRP after the onset of infection, which also correlates with the severity of the infection[4, 8, 14, 21]. In our study, IL-6 levels peaked within 24 h postoperatively and continued to decline over the following 5 days, significantly higher in the PP group than in the Non-PP group (P < 0.001).

WBC, PCT and CRP has been widely tested to detect postoperative pneumonia, but its effectiveness is still controversial[8, 16, 22]. In line with the findings of Jukic et al. we also concluded that WBC alone were not effective in diagnosing early pneumonia after cardiac surgery[12]. Although continuous measurement of PCT may be useful in the diagnosis of PP[23], PCT was also significantly elevated in systemic inflammatory responses syndrome due to cardiac surgery, which may lead to the reduced diagnostic validity[24, 25]. We found that PCT levels showed a difference between the two groups from the first postoperative day (p = 0.02), but with insufficient diagnostic efficacy (AUC < 0.7). CRP is another commonly used marker to determine infection or inflammatory response. We found that CRP showed a significant difference between the two groups from POD2 (P < 0.001) and that CRP on POD3 was a good indicator of PP (AUC:0.77), but multivariate analysis showed that CRP was not an independent risk factor.

Our study showed that the absolute value of IL-6 was more sensitive in the diagnosis of early PP than that of PCT and CRP, WBC. IL-6 may help clinicians determine a patient's risk of developing pneumonia early and these patients can be more targeted for further monitoring and treatment with antibiotics.

### Limitations

Some limitations should be considered in this study. First, this is an observational analysis, and the results support association, not necessarily causation. Second, the data comes from a single center, and agency-specific variables may have influenced the current results. Third, the bronchoalveolar lavage can cause inflammatory response, which may impact the results. Therefore, our results must be further validated by other multicenter studies.

## Conclusion

Elevated IL-6 levels on POD2 as well as smoking, drinking, hypertension, prolonged mechanical ventilation and ICU stay were all independent risk factors for PP. IL-6 levels on POD2 may serve as a promising indicator in predicting PP, better than WBC, PCT and CRP.

## Data Availability

The datasets analysed during this study are obtainable from the corresponding author upon reasonable request.
